# A quantum trust and consultative transaction-based blockchain cybersecurity model for healthcare systems

**DOI:** 10.1038/s41598-023-34354-x

**Published:** 2023-05-02

**Authors:** Shitharth Selvarajan, Haralambos Mouratidis

**Affiliations:** 1 Post Doc Researcher (IADS), University of Essex, Colchester, UK; 2 Department of Computer Science, Kebri Dehar University, Kebri Dehar, Ethiopia; 3 Institute for Analytics and Data Science (IADS), University of Essex, Colchester, England, UK

**Keywords:** Health care, Engineering

## Abstract

Many researchers have been interested in healthcare cybersecurity for a long time since it can improve the security of patient and health record data. As a result, a lot of research is done in the field of cybersecurity that focuses on the safe exchange of health data between patients and the medical setting. It still has issues with high computational complexity, increased time consumption, and cost complexity, all of which have an impact on the effectiveness and performance of the complete security system. Hence this work proposes a technique called Consultative Transaction Key Generation and Management (CTKGM) to enable secure data sharing in healthcare systems. It generates a unique key pair based on random values with multiplicative operations and time stamps. The patient data is then safely stored in discrete blocks of hash values using the blockchain methodology. The Quantum Trust Reconciliation Agreement Model (QTRAM), which calculates the trust score based on the feedback data, ensures reliable and secure data transfer. By allowing safe communication between patients and the healthcare system based on feedback analysis and trust value, the proposed framework makes a novel contribution to the field. Additionally, during communication, the Tuna Swarm Optimization (TSO) method is employed to validate nonce verification messages. Nonce message verification is a part of QTRAM that helps verify the users during transmission. The effectiveness of the suggested scheme has been demonstrated by comparing the obtained findings with other current state-of-the-art models after a variety of evaluation metrics have been analyzed to test the performance of this security model.

## Introduction

Due to their high level of security and guaranteed dependable data transfer and transmission, the cybersecurity models^1–5^ have recently attracted increased attention in many industrial application fields. In general, cybersecuriy^6^ is a type of action or procedure used by several industries to protect data and systems against online attacks. Additionally, it is frequently used by numerous businesses and people to safeguard their private information against criminal activity^7^. Because it is crucial to safeguard patient health information without interruptions, cybersecurity technologies are essential in the healthcare sector^8^. Additionally, cyber-attacks^9,10^ are nefarious or improper behaviors that pose a risk to the confidentiality of healthcare systems' data to benefit themselves. Recent papers^11,12^ assess the everyday growth of cyberattacks and vulnerabilities, which threaten patients' private and confidential information by disrupting routine activities^13,14^. Therefore, it is crucial to design cybersecurity models to safeguard healthcare data systems against cyberattacks^15–17^. The main purposes of applying security approaches are to analyze the various potential ways to identify assaults and provide the necessary solutions to deal with those problems. The integrity, availability, confidentiality, and secrecy qualities are typically satisfied by cybersecurity models to ensure healthcare data security^18–20^.

Additional security criteria should be met to ensure reliable and trusted information sharing in healthcare systems^21,22^. These include requirements for privacy, authenticity, confidentiality, auditing, non-repudiation, secure data transmission, integrity, and access control. Various security techniques are proposed in the current studies to secure the confidentiality and privacy of healthcare systems^23^. In particular, blockchain technology offers sophisticated and thoughtful solutions for handling security issues in healthcare systems. Recent studies^24^ state that research has shown that the blockchain aids in securely storing sensitive data gathered from numerous sources with improved accountability and traceability. Table 1 lists the various security issues and the suitable blockchain solutions for each.

**Table 1:** Different types of security problems in the healthcare systems.

References	Security Problems	Blockchain Solutions	Merits
^25^	Single-point-of-failure and Privacy leak	It supports some access controlling and key management solutions for privacy preservation	1. Guaranteed data security 2. Accountability
^26^	Real-time patient monitoring	Consortium-managed solution	1. Data availability 2. Immutability 3. Secrecy
^27,28^	Privacy issues and attacks	Data encryption and access control mechanisms	1. Privacy 2. Confidentiality 3. Enabled secured data search
^29^	Data breach and malicious access	Customized solutions, and index-based searching	1. Reliable searching 2. Traceability 3. Integrity
^30^	Data sharing and Quality of Service (QoS)	Smart and secured blockchain solutions	1. Privacy protection 2. Efficient message delivery 3. Identity management

Based on the detailed review^31,32^, it is analyzed that the conventional works encountered some of the following potentially difficult issues:

- Maintaining data security and privacy in the electronic medical record system remains a key issue that must be resolved.
- Key pair management is also considered a big issue in healthcare security systems.
- Data security is impacted by ineffective data validation and verification procedures.
- Due to the heterogeneity of medical data maintained in the cloud server, an appropriate and efficient architecture is required to enable trusted and valid information exchange/communication.
- Because of the exponentially increasing number of patients, scalability and availability are also considered major issues.

**Figure 1:**
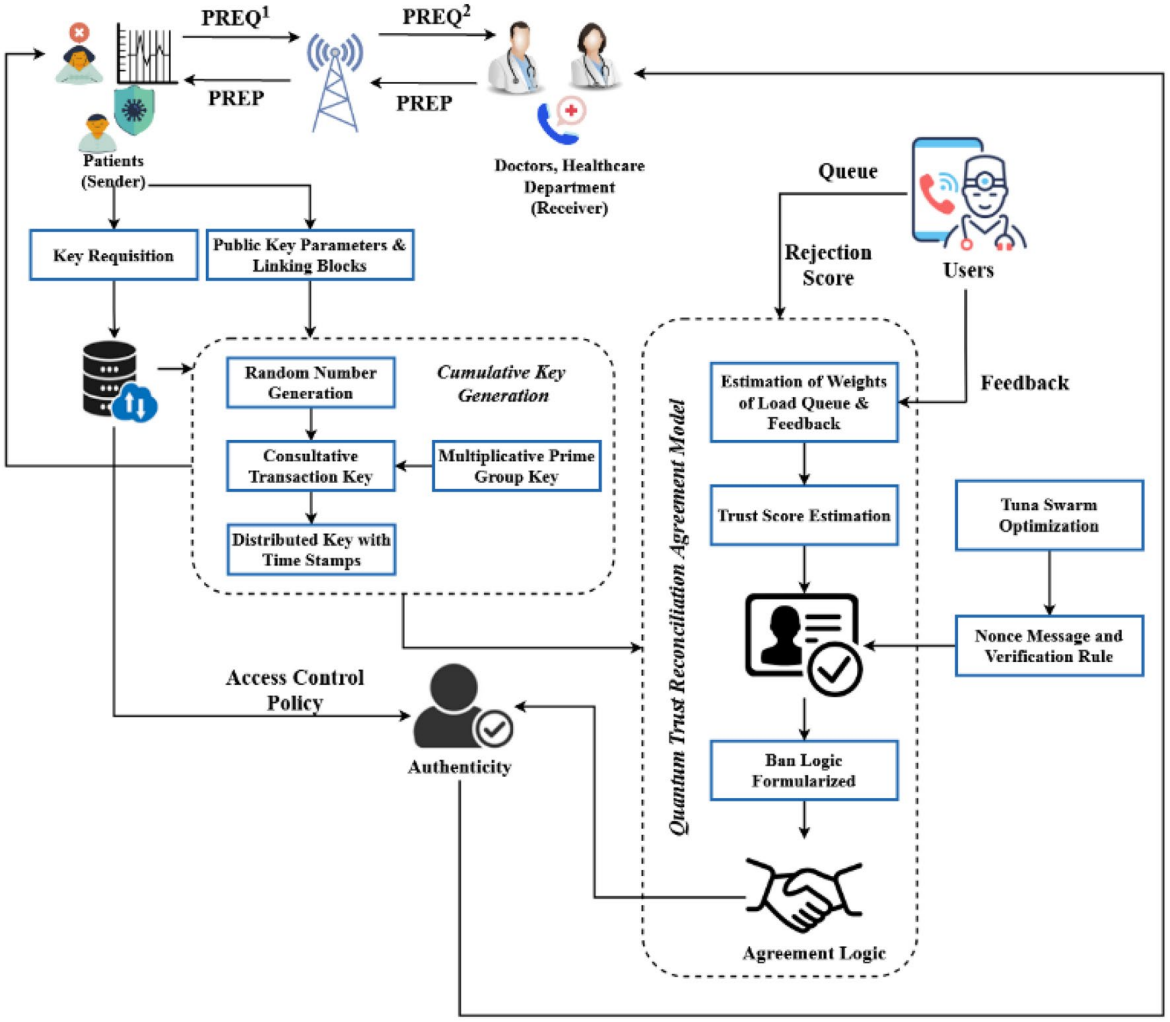
Overall architecture model of the proposed system.

Therefore, this work proposes developing an advanced and novel security model for increasing the security of healthcare systems. Here, the concepts of blockchain, consultative key generation, BAN logic, feedback analysis, and trust score calculation are combined with increasing the security of patient information and data. The consultative key is created by combining the multiplicative prime group key with the random number generated during key generation. Any cryptographic system faces the challenge of efficient and secure key management. Any method an intruder employs to discover the keys will enable them to pilfer everything from the targeted system. Consequently, key administration is one of the most crucial components of a cryptographic system. This work seeks to implement efficient key management in healthcare systems to enable secure and reliable data transmission. In the proposed work, we have formed a decentralized blockchain architecture for assuring security and privacy in healthcare systems. As shown in Fig 1, the patient transmits the request to the healthcare management, and they respond to the requested patient according to their authenticity and trust. Here, the main purpose of using this blockchain technology is to enable a secure transfer of patients’ medical records, to strengthen the healthcare data defenses, to manage the medicine supply chain effectively and, to provide assistance to healthcare researchers to unlock genetic code. During this process, the patient-user can request a key from the database for authentication, where the cumulative key generation model is used to create the key. After that, the QTRAM based blockchain model is deployed to validate the trust of the requested patient user according to the trust score and nonce message generated by using TSO algorithm. The feedback analysis is then carried out by counting the instances of numerous rejection requests, IP address rejection requests, website rejection requests, and undesirable rejection requests. These rejections are used to evaluate if the trust score is low or high; if it's low, the data transaction is forbidden. Additionally, the BAN logic, which upholds the regulations for ensuring a reliable information flow, is also implemented in this system.

The significant contributions and objectives of the proposed work are as follows:

- This work aims to perform an effective key management in healthcare systems for enabling a reliable and secured data transmission.
- To create the Consultative Transaction Key Generation and Management (CTKGM) scheme to bolster patient privacy and medical data security.
- The Quantum Trust Reconciliation Agreement Model (QTRAM), which integrates blockchain technology, is designed to ensure accurate data sharing and communication between patients and the healthcare system.
- The Tuna Swarm Optimization (TSO) algorithm performs nonce message verification to confirm the legitimacy of patient users. This algorithm delivers the best result based on user needs.
- Both optimization and classification technique performance has been validated using performance metrics.

In the proposed system, a combination of CTKGM, QTRAM, TSO, and BAN logic techniques are used to construct the security framework for the healthcare system. The CTKGM technique is applied to generate the key according to the public parameters of the patients, where the random number generation, transaction key generation based on a multiplicative prime number, and time stamp value generation are performed while generating the key for the requested user. Then, the QTRAM technique is implemented to validate the authenticity of the requested user based on the feedback analysis and trust score values. Once the trust score is estimated, the nonce message is randomly generated using the TSO algorithm, which helps identify the trusted users. Consequently, the ban logic is formulated according to the trust score; based on this, the user is authenticated for further access. Estimating the level of trust is crucial to the security framework for determining the authenticity and validity of the users. Typically, the estimation of trust is based on the data and access credentials of the users. It could be extremely beneficial for restricting unauthorized or unauthenticated access to healthcare systems. Since there is an increased possibility of data leakage in healthcare applications, such as the exploitation of patients' medical data, healthcare data defenses, medicine data, and genetic code, there may be an increase in data leakage risks. Consequently, establishing trust could significantly enhance data security. To this end, the proposed work intends to implement the QTRAM technique for validating the authenticity of users based on their trust score, which is estimated using feedbacks, nonce messages, and ban logic. This type of trust estimation greatly strengthens the healthcare system's security against unauthorized users. The remaining sections of this work can be divided into the following categories: Section “Related works” discusses the features and operational principles of the current cybersecurity models relating to authentication, trust estimation, encryption, decryption, and access control. The study process is then thoroughly described in Section “Methods”, along with a complete flow diagram and algorithmic examples. The performance analysis of existing and proposed security models used in the healthcare systems are validated and compared in Section Results and discussion. In Section “Conclusion”, the entire paper is summarized together with its future scope.

## Related works

Feng et al.^33^ implemented a lightweight collaborative authentication mechanism for enhancing the security of Smart Electronic Health Records (SEHRs). This framework was developed based on two-party communication, where the private key was protected with ensured fairness between the patient and doctor. It has the significant benefits of increased efficiency, guaranteed key protection, and secured communication between the parties. However, it limits the complex problems in key construction and requires high communication costs. Qiu et al.^34^ developed an advanced Medical Cyber-Physical Systems (MCPS) architecture using the selective encryption mechanism to ensure secure health data sharing. Data encryption and decryption processes were carried out here to strengthen the privacy preservation of health data using the fragmentation concept. However, it has problems due to slow computation and a lack of data security. An anonymous certificateless encryption mechanism was used by Ullah et al.^35^ to guarantee the forward data security of Internet of Health Things (IoHT) systems. The random oracle concept and the Hyper Elliptic Curve (HEC) were combined to create this framework. Its primary benefits include low communication and processing costs and good data security. To increase the privacy and security of health data, Wang et al.^36^ combined a blockchain concept with a graph convolutional network (GCN). It strengthened data security and improved intrusion detection performance by incorporating Blockchain technology. Due to inadequate training data models, this detection framework was still insufficient, reducing the system's effectiveness. Sun et al.^4^ proposed a fine-grained access restriction mechanism to complete the optimal vector transformation and improve health-IoT data systems' security. Access control mechanisms have been used for online and offline data encryption procedures. Reduced risks, costs, and information leakage were the main advantages of this architecture. However, it has significant drawbacks, with high authentication delays, needing more storage, and decreased efficiency. Zouka and Hosni^37^ intended to utilize Artificial Intelligence (AI) technology to enhance healthcare systems' data security. The major goal of this research was to use a fuzzy logic decision support system to safely identify patients' health states. Further, by efficiently producing the membership functions, it achieved decreased data transfer time, verification time, and overhead. However, the overall system's performance suffered due to this effort's failure to lower the cost of data transfer and storage. A thorough overview of numerous data privacy and security challenges in cyber medical systems was presented by Habibzadeh et al. in their study^38^. To reduce the vulnerabilities of health data-sharing systems, various encryption and decryption algorithms were examined in this article. Other security criteria for safeguarding healthcare data systems from malicious individuals were studied by Ahmed et al.^33^. It contains the compromise indications, intelligence drive, penetration testing, vulnerability assessment, and resilience metric.

Banerjee et al.^39^ introduced a new quantum blockchain methodology incorporated with the weighted hyper-graph model for data security. This paper aims to formulate a novel decentralized security protocol with cost-effectiveness blockchain technology. A collection of extremely entangled multipartite quantum states called the quantum hyper-graph states are built on the hyper-graph in mathematics. Li et al.^40^ introduced an efficient quantum blockchain methodology to protect quantum computers from malicious attacks. Here, the Quantum Delegated Proof of Stake (QDPoS) model enables fast decentralization with increased efficiency. Khan et al.^41^ investigated the major effects and opportunities of using blockchain and AI in IIoT systems. Zhang et al.^42^ designed an Elastic Batched Byzantine Fault Tolerance Consensus Protocol (EB-BFT) to improve the security of business applications. The number of consensus nodes is dynamically altered by this approach, which recognizes low-performance nodes and prevents them from taking part in consensus. After confirming that the transactions provided by clients are legitimate, an approach for dynamically expanding the amount of batched transactions in consensus is suggested by the authors. Tian et al.^43^ constructed a Dependable Committee Consensus Protocol (DCCP) for ensuring reliability and security. In this model, the mining process and node reliability value are integrated together to choose the most relevant nodes for committee operation. The major advantage of this protocol is, it inhibits the entry of unauthorized nodes in the network with guaranteed network throughput.

Mierzwa et al.^44^ offered a novel cyber assessment approach to improve the security of international healthcare systems. Various cybersecurity risk assessment management principles for healthcare systems were proposed in this paper. Alazab et al.^45^ investigated the effectiveness of different federated learning models for cyberattack detection. This article provides a concise summary of how federated learning methods are used in a variety of industries, including personal healthcare, social media, traffic management, and smart cities. A Blockchain-based deep learning model was implemented by Rathore and Park^46^ to increase the security of cyber-physical systems. Jan et al.^47^ utilized a lightweight authentication approach to enhance the functionality of cyber-physical systems. Also, a Hidden Markov Model (HMM) has been used in this study to identify patients to prevent information leaking. Additionally, mutual authentication and session key management mechanisms were established using BAN logic. A fog-based data aggregation approach was suggested by Guo et al.^48^ to improve the privacy and secrecy of e-healthcare systems. The key generation, addition, scalar multiplication, encryption, and decryption processes were carried out using the Symmetric Homomorphic Encryption (SHE) scheme. The factors of batch verification, data flexibility, integrity, authentication, and privacy protection were also achieved. A low-memory symmetric key generation algorithm was created by Coelho et al.^49^ to enable secure data transmission in IoHT devices. In this case, a secret key agreement protocol was developed to securely carry out the data exchange. The privacy and security issues in the e-health management system were examined by Chenthara et al.^50^. It recommends using cryptographic and non-cryptographic models to address such problems. Based on this analysis, it is determined that the traditional models are heavily concentrated on enhancing healthcare data security based on authentication, access control, key validation, and communication assured by trust. However, it has difficulty in generating keys due to high complexity issues, takes more time, costs more to store, and is less flexible.

The majority of blockchain techniques for enhancing the security of healthcare systems have been implemented in extant works, according to the available literature. Some of the techniques that face the greatest obstacles in terms of time consumption, processing delays, and lack of dependability. Consequently, the proposed work aims to implement a lightweight and efficient blockchain methodology for healthcare security, where effective key management and blockchain techniques are implemented to restrict authenticated access to healthcare systems. Using the CTKGM mechanism, the appropriate key generation is performed based on the public parameters and attributes of the user. In addition, the QTRAM model is used to validate the user's authenticity based on the feedback analysis and trust score. Thus, the combination of the CTGKM-QTRAM model ensures high security in healthcare systems while reducing processing time and delay.

## Methods

This section provides a detailed explanation of the suggested methodology, along with an overview of its design and examples of its algorithms. Many blockchain techniques are created in traditional efforts to boost the security of healthcare systems. However, the issues of poor data handling, rising expenses, and slowed processing speed continue to exist. The proposed work uses an advanced blockchain technique to improve the security and confidentiality of healthcare data. The primary goal of this effort is to enable safe data transfer in healthcare systems utilizing cybersecurity techniques based on blockchain technology. To build a trustworthy data transmission between the patients and the healthcare system, an upgraded security architecture is designed based on this goal.

From the existing works, we have studied the different types of intrusions or cyber-attacks that highly disrupts healthcare systems in recent times. Also, the effects of security threats and vulnerabilities are studied according to their characteristics and functions. In addition, some of the possible security solutions are examined from these existing works. Based on this analysis, a clear overview about cyber-security in healthcare applications is studied, and also it is more helpful for us to implement the blockchain-based security model for healthcare systems. Blockchain is one of the most recent technologies widely deployed in different fields to guarantee data security and confidentiality. A distributed ledger technology enables reliable communication in the environment using cryptographic primitives. Specifically, it gained significant attention in the healthcare domain due to its immutability, persistency, privacy, and decentralization features. With modern internet technology, healthcare services are moved to online mode, but it is highly susceptible to more security issues like interoperability, security breaches, scattered data, and scalability. In recent days, healthcare systems use the centralized database systems for storing patient’s health information. Typically, the distributed storage system is more expensive in cost and time; hence, medical experts highly prefer centralized storage systems for the health data management field. However, it is also a memory-consuming task since the healthcare data must be encrypted before storing it in the cloud systems. According to the recent reviews, it is analysed that there are various blockchain-integrated healthcare applications are developed in the conventional works. Furthermore, it shows the prominence and applicability of blockchain technology in the healthcare domain field where data privacy, security, and authenticity are mainly concentrated. Also, the majority of the existing security frameworks use the blockchain solutions for protecting the patients’ private information from the unauthorized access. Yet, the existing studies facing the major challenges in terms of high complexity in system chain, storage overhead, large processing time, and low speed. Therefore, the proposed work motivates to implement a lightweight as well computationally effective blockchain model for healthcare security. To accomplish this objective, a Consultative Transaction Key Generation and Management (CTKGM) integrated with Quantum Trust Reconciliation Agreement Model (QTRAM) based blockchain model is deployed in this work. The proposed architecture employs the distributed blockchain model that is more suitable for the next-generation healthcare application systems. Also, a lightweight access controlling mechanism is developed using the hyperledger blockchain methodology for the healthcare systems. In this framework, a common data sharing platform has been utilized for connecting the disjoint stakeholders in the healthcare sector. The key benefits of this framework are economic-friendly, optimal memory consumption, guaranteed security, trusted communication and data sharing.

Given this, the system uses intelligent key creation, trust estimation, and optimization approaches. The suggested model uses a private blockchain for the hospital setting, limiting access to the data to those who are permitted. Additionally, it effectively increases the processing of remote monitoring and protects patient information, diagnostic details, medication details, etc. The proposed cybersecurity model in healthcare systems is depicted in Fig. 1 and includes the following modules:

- Consultative transaction key generation and management
- Quantum trust reconciliation agreement model
- Tuna swarm optimization

According to this architecture, the patient serves as the user or source of the transmitter, and the healthcare organization serves as the receiver. The network manager, on the other hand, acts as a middleman between the data sender and recipient. In order to obtain their license to begin the data transfer, the patient-user can first register their information with the hospital server. The network administrator generates a unique private and public key pair along with the user's license when they submit a registration request to the server using the Consultative Transaction Key Generation and Management tool (CTKGM). This study employs a novel approach for producing the unique key pair based on the procedures of generating random values, performing multiplicative operations, and distributing the keys according to timestamp values. The recipient could not have access to the personal health information once the session terminates due to the time stamp value. The data is encrypted and saved in the server using the blockchain technique after the keys are generated. In the proposed security framework, the Elliptic Curve Cryptography (ECC) model is used to generate the private and public key pairs. Since, the ECC has the better ability to protect the data against unauthorized access, when compared to the other asymmetric encryption mechanisms. The specific advantages of using the ECC approach are listed below:

- Fast key and signature generation
- Authenticated key exchange
- Easy to deploy
- Reduced latency
- Low complexity

Therefore, the proposed work uses the ECC technique for key generation, and is suitable for both quantum and classical computing systems. This methodology divides the data into blocks that are then recorded in hash values. Additionally, it improves the protection of data against unauthorized users. The Quantum Trust Reconciliation Agreement Model (QTRAM) is used to set up the secure sharing of data between the server and receiver at the time of transmission. Before data transmission, the feedback data is evaluated, and this agreement model is combined with models for estimating trust scores and BAN logic. With the use of tools like IP address identification, website identification, and the denial of undesired repetitive requests, feedback analysis is primarily used to identify the requested users, whether they are trusted or not. There is no need to check the rejection potential with the server because it has been easily established using this information. Moreover, the nonce message is verified using the Tuna Swarm Optimization (TSO) algorithm, preserving the confidentiality of the data receiver. The nonce message, which is more frequently used in cryptographic communications, is typically some random integer generated for verification purposes. As a result, the rules employed to provide guaranteed information transmission between the entities are expressed as the BAN logic. The trust score is estimated based on the multiple rejections of the service request; a high number of rejections will result in a low trust score. By combining the mechanisms for feedback analysis, BAN logic, and trust score estimation, the proposed QTRAM model significantly improves data security. High security, dependable data transmission, high operating efficiency, and minimal time consumption are the main advantages of this work. In the proposed security model, the consultative key generation mechanism is used to generate the keys for user validation and authentication according to their public parameters. Initially, the patient user give the key requisition for authentication and access, during this process the random number generation, transaction key generation based on multiplicative prime number, and time stamp values are generated for creating key. Once, the user key is generated using CTKGM, it can be further validated by the QTRAM model for user authentication. Here, the private and public key pair are generated along with the hash value, and the user transmits it to the blockchain for storage. According to the current timestamp value and duration, the key is validated for further user transactions.

For instance, consider a healthcare system, the patient users can give request to the healthcare department for medical advice and data access. So, the user should be registered with the cloud server at first with their public key parameters, and if the user is already registered, he/she must be authenticated before data access. During this process, the user can give key requisition from the database, where the CTKGM technique can generate the key according to the user public key parameters. Once the key is generated, it is sent to the blockchain for storage, where the trust score estimation, nonce message verification, and ban logic validation are performed to validate the trust of user. Further, the user can be authenticated with the access control policy for data access. In this scenario, the key management is performed for assuring both data security as well user authentication.

**Figure 2:**
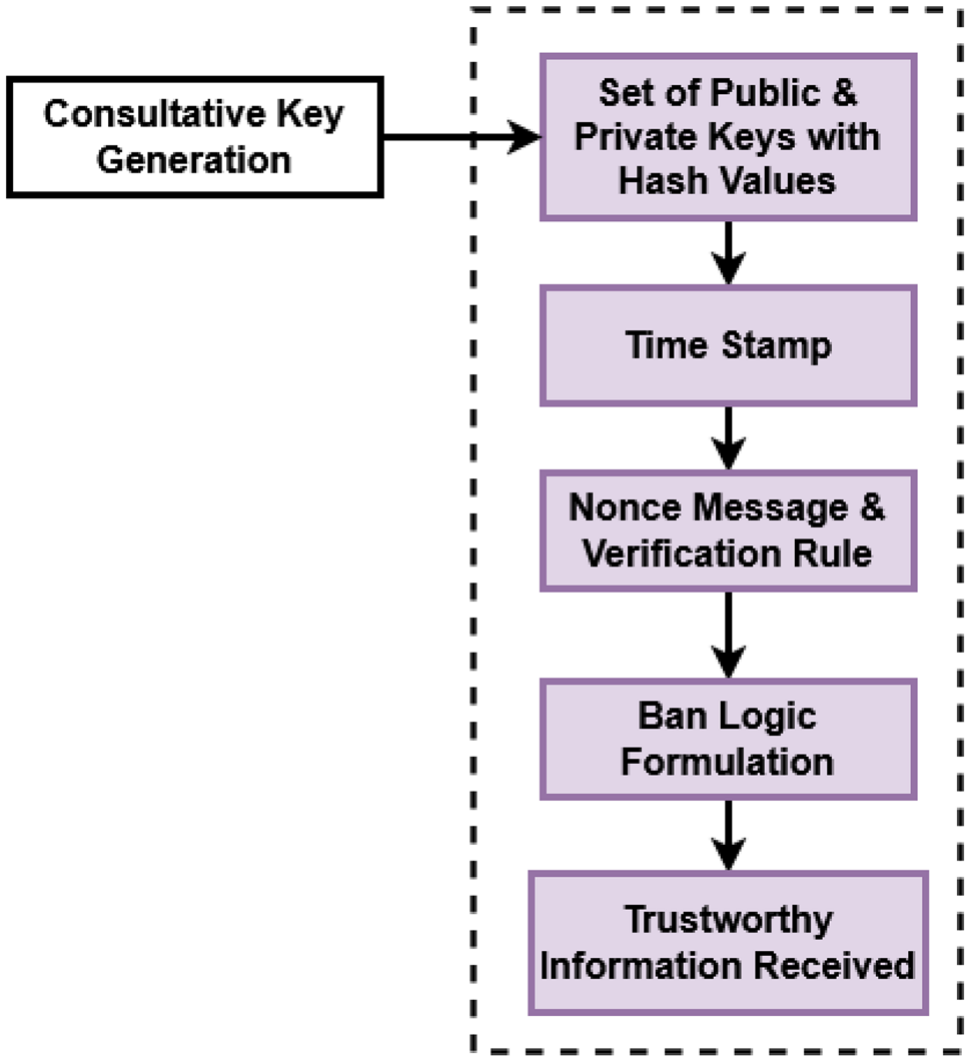
Structure of blockchain.

Figure 2 represents the structure of blockchain model used in the proposed framework, which stores the information private & public keys with the hash values, time stamp, nonce message with the verification rule, Ban logic, and trustworthy information received. Based on these information, the user verification and authentication are carried out in the proposed framework.

### Consultative transaction key generation and management (CTKGM)

**Figure 3:**
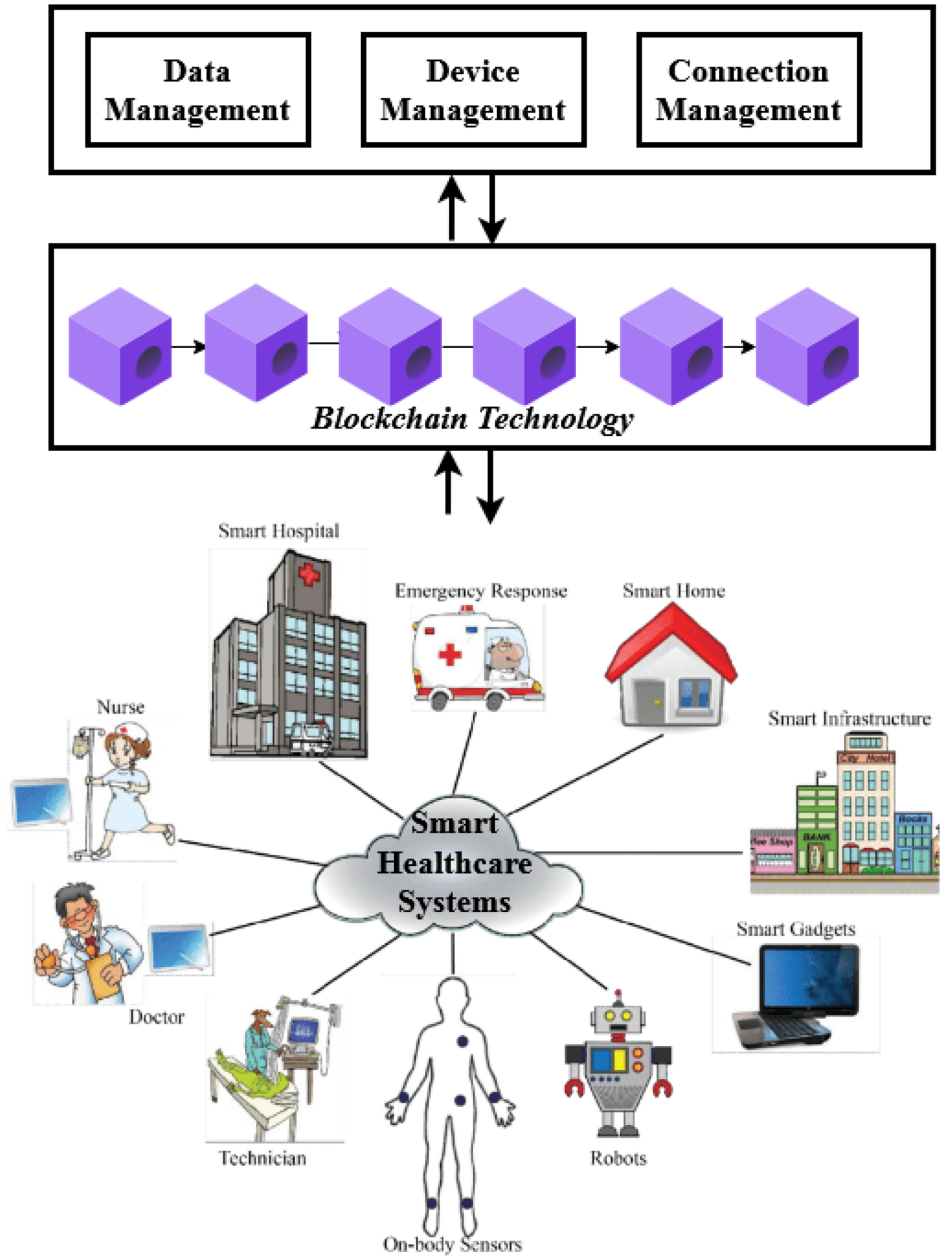
Layered architecture model of cybersecurity in healthcare systems.

Figure 3 explains the layered architecture model of cybersecurity in healthcare system. In this model, system parameters such as the total number of patients, the number of active patients, authentication data, the personal health information of each patient, and the unique identity of each patient are selected by the Hospital Server (HS), which is the top layer. This layered architecture is mentioned in Fig. 2 and the necessary parameters and descriptions are provided in Table 2.To obtain their specific license, which can subsequently be used to authenticate users as valid or not, new patients at the hospital must register their personal and medical information on the hospital server. The hospital server/network manager, who serves as an intermediary between the sender and receiver of data transmission, can be asked to register during this procedure by the PU. The network manager is in charge of creating the key pair for all users connected to them. Let's say the server has the requested patient's registration. The unique id along with the certificate is provided to that user, which comprises the information of the unique hospital server identity $${(\tau }_{i}$$), the unique identity of the PU belongs to the hospital server ($${\delta }_{j}$$), a permanent public key of the server ($${p}_{k}^{s}$$), and signature along with the private key of PU ($${p}_{p}^{s}PU)$$. Using this information, the PU sends the encrypted data to the server for storage using the blockchain methodology. In this case, the secret information (such as health information or personal data) is transferred from the patient to the hospital, and the private key is utilized to generate the signature. Because of this, smart contracts have been developed to encrypt patient data kept in blockchain form, so if the recipient wants to access the data, it needs to be individually verified for each user using a different ID. The network manager created the following format for the certificate for the registered PU:

1 $$P{U}^{L}=({\tau }_{i}||{\delta }_{j}||{p}_{k}^{s}||{sign}_{ {p}_{p}^{s}})$$

where $P{U}^{L}$ represents the license of PU. When the registered patient wants to share their information with the receiver in the same domain, encryption keys are required for sharing and accessing the data. During encryption, the $P{U}^{T}$ generates the seed point $\rho$ based on the random number selection of $\rho \in \left(0, 1....,\mathrm{ p }- 1\right)$. Then, the data can be encrypted with the value of $\rho$ and the public key of the PU belongs to the ${\mathrm{PU}}^{\mathrm{X}} ({\delta }_{PU})$, where ${\mathrm{PU}}^{\mathrm{X}}$ indicates the number of all patients. After that, the PU sends the license along with the above message for verifying itself with ${\mathrm{PU}}^{\mathrm{X}}$. Based on this way, the *M* number of public and private key pairs are generated with the hash values $\varphi$ for all the patients in the hospital sector, which is done by using the one-way hash chain model. Consequently, the encrypted data can be stored in the server using the Blockchain model, which is in the form of ${B}^{N}(\rho )$. Then, the current timestamp value and duration can be determined for the respective data with the information of how long the key set will be valid corresponding to the $P{U}^{T}$.

**Table 2:** List of parameters and descriptions.

Parameters	Descriptions
PUs	Patient users
${\tau }_{i}$	Unique hospital server identity
${\delta }_{j}$	Unique identity of the PU
${p}_{k}^{s}$	Permanent public key of the server
${p}_{p}^{s}$	Signature using the private key
$P{U}^{L}$	License of Patient user
$sign$	Signature
$HS$	Hospital server
$\rho$	Seed
*M*	Number of public and private key pairs
$\varphi$	Hash values
${\mathrm{PU}}^{\mathrm{X}}$	Number of all patients
${B}^{N}(\rho )$	Encrypted data stored in the server
${t}^{k}$	Time interval
${\mathfrak{R}}_{t}$	Cipher text of random number
$\Delta \tau$	Maximum tolerable time interval
$\omega$	Value of ω is obtained from the corresponding PUs
$x and y$	Pseudo-random numbers
${a}^{1}$ and ${a}^{2}$	Optimization constants
${t}^{1}$ and ${t}^{2}$	Timestamp of sender and receiver parties
${u}^{1}$	Sender i.e. patient user
${u}^{2}$	Receiver i.e. hospital server
${M}^{1}$	Generated message at the time of data transmission
${M}^{2}$	Generated message at the time of receiving data
${T}_{1}$,${T}_{2}$,${T}_{3}$ and ${T}_{4}$	Targets
${P}_{ub}$	Secret information shared between the users
SK	Shared key
${P}_{S}$	Population Size
$Ma{x}_{t}$	Maximum iteration
${O}^{T}$	Optimization constant
${X}_{i}^{T}$	Random population of tunas
$a$ and $z$	Free parameters
${X}_{best}^{ T}$	fitness value
${X}_{i}^{t+1}$	Population position
R	Random number
${X}_{rand}^{ T}$	Randomly generated the reference point

If the $P{U}^{T}$ wants to connect with the ${HS}^{R}$ for accessing or sharing the data information, it is more required to prove its authenticity to the ${HS}^{R}$, then only the session key can be generated for further communications. At this time, the $P{U}^{T}$ can initiate the communication with the interval of ${t}^{k}$, $0 \le k < M$ by directly sending the message to the ${HS}^{R}$, which is represented as follows:

2 $$Pk{p}_{k}^{s}=\prod_{n=0}^{k}{B}^{n}( \rho )$$

Then, the cipher text of the random number ${\mathfrak{R}}_{t}, (0<{\mathfrak{R}}_{t}<p-1)$ can be generated by using the public key of ${HS}^{R}$, which is in the form of $[{E}_{{{p}_{p}^{s}}_{{HS}^{R}}}({\mathfrak{R}}_{t})]$ with the time stamp value ${T}^{k}$ and certificate $P{U}^{L}$. The following model represents the format of data transmission from the patient to the hospital server,

3 $${P{U}^{T}}\stackrel{P{U}^{T}{p}_{k}^{s}, license,{T}^{k},{E}_{{{p}_{p}^{s}}_{{HS}^{R}}}({\mathfrak{R}}_{t})}{\to } {HS}^{R}$$

Once, the ${HS}^{R}$ received the above message, it requires to verify the following condition:

4 $$T^{T} - T^{R} < \Delta \tau$$

where ${T}^{T}$ is the current system time at $P{U}^{T}$, and $\Delta \tau$ is the maximum tolerable time interval. If it is valid, the correctness of the certificate obtained from cap P, cap U to cap T is verified to ensure security. If the certificate matches, the ${S}^{R}$ computes the following model:

5 $$P{U}^{T}{p}_{i}^{s},\left(k+1\right)\le i\le M$$

Also, the ${HS}^{R}$ verifies the authenticity of $P{U}^{T}$ for validating the following condition:

6 $$b\left({\omega }^{*}\right)=\omega$$

7 $${\omega }^{*}=P{U}^{T}{p}_{k}^{s}\prod_{n=0}^{k}{B}^{n}( \rho )$$

where $\omega$ can be obtained from the corresponding $PUs$. If the condition is not satisfied, the ${HS}^{R}$ can reject the request and report to the PU; otherwise, it decrypts the message as shown below:

8 $$P{U}_{{p}_{p}^{s}\_P{U}^{T}} \left[{E}_{{{p}_{p}^{s}}_{P{U}^{R}}}\left({\mathfrak{R}}_{t}\right)\right]={\mathfrak{R}}_{t}^{*}$$

Also, the receiver uses the private key and selects the random number based on ${\mathfrak{R}}_{t}, (0<{\mathfrak{R}}_{t}<p-1)$. After accessing the data, the ${HS}^{R}$ sends the reply message with the information of $P{U}^{T}{p}_{l}^{s}\prod_{n=0}^{l}{B}^{n}( \rho ),$ $\left[{E}_{{{p}_{p}^{s}}_{P{U}^{R}}}\left({\mathfrak{R}}_{r}\right)\right]and h({\mathfrak{R}}_{r}||{\mathfrak{R}}_{t}^{*})$ to the corresponding ${PU}^{T}$ as shown in the following format:

9 $${P{U}^{T}}\mathop{\longleftarrow}\limits^{{P{U}^{T}{p}_{l}^{s}, license,{T}^{k},{E}_{{{p}_{p}^{s}}_{P{U}^{T}}}({\mathfrak{R}}_{r})}} {HS}^{R}$$

When the $P{U}^{T}$ receives the reply message, it follows the same process for verifying the identity of ${HS}^{R}$. If it is valid, the condition $P{U}_{{p}_{p}^{s}\_{HS}^{R}} \left[{E}_{{{p}_{p}^{s}}_{P{U}^{T}}}\left({\mathfrak{R}}_{r}\right)\right]={\mathfrak{R}}_{r}^{*}$ is computed and verified as shown below:

10 $$h({\mathfrak{R}}_{r}|\left|{\mathfrak{R}}_{t}^{*}\right)=h({\mathfrak{R}}_{t}^{*}||{\mathfrak{R}}_{r})$$

Then, the $P{U}^{T}$ sends the acknowledgement to the ${HS}^{R}$ and, finally both $P{U}^{T}$ and ${HS}^{R}$ computes the session key for establishing further communications as represented below:

11 $$h(P{U}^{T}{p}_{k}^{s}|\left|{\mathfrak{R}}_{r}|\left|{\mathfrak{R}}_{t}\right||P{U}^{T}{p}_{l}^{s}\right)$$

This system ensures secured data sharing between the patients and the healthcare system with reliable communication.

### Quantum trust reconciliation agreement model

The Quantum Trust Reconciliation Agreement Model (QTRAM) is used in this framework primarily to build safe communication between patients and the healthcare system based on feedback analysis and trust value. It is more important than ever in every data communication system to evaluate user feedback before transmission. As a result, the QTRAM is used in this study, where the trust value is calculated using user feedback as well as add-on data (such as rejection score). For this purpose, the BAN logic has been utilized that facilitates secured communication by constructing a set of rules, which are in the form of $\frac{M}{N}$, where M indicates correct and N indicates incorrect. Typically, the BAN logic has a set of regulations on message freshness, meaning, jurisdiction, and reception. Here, the message verification rule ($Rul{e}_{1}$) is used to validate the message between the communicating parties such as the patient and the hospital server. It works based on the following logic: the $P{U}^{T}$ considers that the shared key between the patient user and hospital server is in the form of $h(P{U}^{T}{p}_{k}^{s}|\left|{\mathfrak{R}}_{r}|\left|{\mathfrak{R}}_{t}\right||P{U}^{T}{p}_{l}^{s}\right)$, and the PU received the message in the form of ${\{M\}}_{h(P{U}^{T}{p}_{k}^{s}|\left|{\mathfrak{R}}_{r}|\left|{\mathfrak{R}}_{t}\right||P{U}^{T}{p}_{l}^{s}\right)}$ encrypted with $h(P{U}^{T}{p}_{k}^{s}|\left|{\mathfrak{R}}_{r}|\left|{\mathfrak{R}}_{t}\right||P{U}^{T}{p}_{l}^{s}\right)$. It is mathematically represented as follows:

12 $$\frac{P{U}^{T}|\equiv P{U}^{T}\mathop{\longleftrightarrow}\limits^{h(P{U}^{T}{p}_{k}^{s}|\left|{\mathfrak{R}}_{r}|\left|{\mathfrak{R}}_{t}\right||P{U}^{T}{p}_{l}^{s}\right)} {HS}^{R}, P{U}^{T}{\{M\}}_{h(P{U}^{T}{p}_{k}^{s}|\left|{\mathfrak{R}}_{r}|\left|{\mathfrak{R}}_{t}\right||P{U}^{T}{p}_{l}^{s}\right)}}{P{U}^{T}\left|\equiv {HS}^{R} \right| \sim \{M\}}$$

Consequently, the nonce-verification rule $Rul{e}_{2}$ is formed, if the $P{U}^{T}$ believes that $\{M\}$ is new, and also it trusts the hospital server has $\{M\}$. Moreover, the protocol messages are emphasized with the help of BAN logic, because it has a unique set of logical symbols. Also, it is more essential to use the formal logic for protocol security analysis, where the protocol is described with the unique symbols of BAN logic as shown below:

13 $${M}^{1} {u}^{2} \leftarrow (x,{a}^{1},{t}^{1},{u}^{1}\mathop{\longleftrightarrow }\limits^{h(P{U}^{T}{p}_{k}^{s}|\left|{\mathfrak{R}}_{r}\left|\left|{\mathfrak{R}}_{t}\right|\right|P{U}^{T}{p}_{l}^{s}\right)}{u}^{2})$$

14 $${M}^{2} {u}^{1} \leftarrow (y,{a}^{2},{t}^{2},{u}^{2}\mathop{\longleftrightarrow }\limits^{h(P{U}^{R}{p}_{k}^{s}|\left|{\mathfrak{R}}_{t}\left|\left|{\mathfrak{R}}_{r}\right|\right|P{U}^{R}{p}_{l}^{s}\right)}{u}^{1})$$

where $x and y$ are the pseudo-random numbers that are used for generating the BAN logic at both the sender and receiver sides. Then, the parameters ${a}^{1}$ and ${a}^{2}$ are the optimization constants obtained from the TSO algorithms, ${t}^{1}$ and ${t}^{2}$ are the time stamp of the sender and receiver parties, ${u}^{1}$ is the sender i.e. patient user, ${u}^{2}$ is the receiver i.e. hospital server, ${M}^{1}$ is the generated message at the time of data transmission, and ${M}^{2}$ is the generated message at the time of receiving data. In this security framework, the main use of the QTRAM protocol is to generate the group key only for the internal members of healthcare systems and by using this key, the security of subsequent communications is ensured. Hence, this work developed the trust reconciliation agreement protocol based on the blockchain methodology, which includes the following security goals ${T}_{1}-{T}_{4}$:

15 $${T}_{1} {u}^{1}|\equiv {u}^{1}\stackrel{h(P{U}^{T}{p}_{k}^{s}|\left|{\mathfrak{R}}_{r}\left|\left|{\mathfrak{R}}_{t}\right|\right|P{U}^{T}{p}_{l}^{s}\right)}{\longleftrightarrow }{u}^{2}$$

16 $${T}_{2} {u}^{2}|\equiv {u}^{1}\stackrel{h(P{U}^{T}{p}_{k}^{s}|\left|{\mathfrak{R}}_{r}\left|\left|{\mathfrak{R}}_{t}\right|\right|P{U}^{T}{p}_{l}^{s}\right)}{\longleftrightarrow }{u}^{2}$$

17 $${T}_{3} {u}^{1}|\equiv {u}^{2}|\equiv {u}^{1}\stackrel{h({HS}^{R}{p}_{k}^{s}|\left|{\mathfrak{R}}_{t}\left|\left|{\mathfrak{R}}_{r}\right|\right|{HS}^{R}{p}_{l}^{s}\right)}{\longleftrightarrow }{u}^{2}$$

18 $${T}_{4} {u}^{2}|\equiv {u}^{1}|\equiv {u}^{1}\stackrel{h({HS}^{R}{p}_{k}^{s}|\left|{\mathfrak{R}}_{t}\left|\left|{\mathfrak{R}}_{r}\right|\right|{HS}^{R}{p}_{l}^{s}\right)}{\longleftrightarrow }{u}^{2}$$

where ${T}_{1}$ and ${T}_{2}$ are the targets, ${u}^{1}$ and ${u}^{2}$ are the users who believe that they have established a shared $h(P{U}^{T}{p}_{k}^{s}|\left|{\mathfrak{R}}_{r}\left|\left|{\mathfrak{R}}_{t}\right|\right|P{U}^{T}{p}_{l}^{s}\right)$ with each other. Then, the targets ${T}_{3}$ and ${T}_{4}$ considers that ${u}^{1}$ and ${u}^{2}$ believes the other party, who already knows the key of $h({HS}^{R}{p}_{k}^{s}|\left|{\mathfrak{R}}_{t}\left|\left|{\mathfrak{R}}_{r}\right|\right|{HS}^{R}{p}_{l}^{s}\right)$ used for communication. The following assumptions have been made for defining the hypothesis condition using this agreement protocol. Let consider ${P}_{ub}$ is the secret information shared between the users ${u}^{1}$ and ${u}^{2}$, and SK is the shared key.

19 $${B}_{1} {u}^{1}|\equiv {u}^{1}\stackrel{{P}_{ub}}{\longleftrightarrow }{u}^{2}$$

20 $${B}_{2} {u}^{2}|\equiv {u}^{1}\stackrel{{P}_{ub}}{\longleftrightarrow }{u}^{2}$$

21 $${B}_{3} {u}^{1}|\equiv {u}^{2}|\equiv {u}^{1}\stackrel{h(P{U}^{R}{p}_{k}^{s}|\left|{\mathfrak{R}}_{t}\left|\left|{\mathfrak{R}}_{r}\right|\right|P{U}^{R}{p}_{l}^{s}\right)}{\longleftrightarrow }{u}^{2}$$

22 $${B}_{4} {u}^{2}|\equiv {u}^{2}|\equiv {u}^{1}\stackrel{h(P{U}^{R}{p}_{k}^{s}|\left|{\mathfrak{R}}_{t}\left|\left|{\mathfrak{R}}_{r}\right|\right|P{U}^{R}{p}_{l}^{s}\right)}{\longleftrightarrow }{u}^{2}$$

By using the formal messages, the logical inference rules are obtained as illustrated below:

23 $$Prof_{1} :u^{2} \left| { \equiv u^{1} } \right|\sim(a^{1} ,t^{1} ,u^{1} \mathop {\longleftrightarrow} \limits^{{h(PU^{T} p_{k}^{s} ||\Re_{r} \left| {\left| {\Re_{t} } \right|} \right|PU^{T} p_{l}^{s} )}} u^{2}$$

From ${b}_{4}$ and a new rule of $Rul{e}_{1}$, the hypothetical sentence $Pro{f}_{2}$ can be obtained:

24 $$Prof_{2} :u^{2} \left| { \equiv u^{1} } \right|\sim\left( {a^{2} ,t^{2} ,u^{2} \mathop {\longleftrightarrow}\limits^{{h(PU^{R} p_{k}^{s} ||\Re_{t} \left| {\left| {\Re_{r} } \right|} \right|PU^{R} p_{l}^{s} )}} u^{1} } \right)$$

Based on $Pro{f}_{1}$ and $Pro{f}_{2}$, the statement $Pro{f}_{3}$ can be inferred:

25 $$Prof_{3} :u^{1} | \equiv u^{1} \mathop \leftrightarrow \limits^{{h(PU^{T} p_{k}^{s} ||\Re_{r} \left| {\left| {\Re_{t} } \right|} \right|PU^{T} p_{l}^{s} )}} u^{2}$$

Based on this agreement logic, the data is transmitted between the patient-user and the hospital server.

### Tuna swarm optimization

The key factor of using the TSO algorithm is performing nonce message verification based on random value generation. It is frequently used in various application systems to solve challenging optimization issues and is typically a meta-heuristic technique. During communication, nonce verification packets are verified using this optimization technique. This model generates a set of default nonce messages for every patient user registered with the hospital server. The system automatically creates nonce messages based on the patient ID, name, and other health information data when a registered user logs in to verify the user's identity. By calculating the ideal fitness value, it individually constructs the default nonce messages for the group of registered users. Each patient-user enrolled on the server may receive a different nonce message depending on the optimal value. Fast convergence speed, the most optimal solution, decreased time consumption, minimal computing complexity, and great efficiency are the main advantages of employing the TSO technique. Below is a representation of the TSO technique's algorithmic steps:
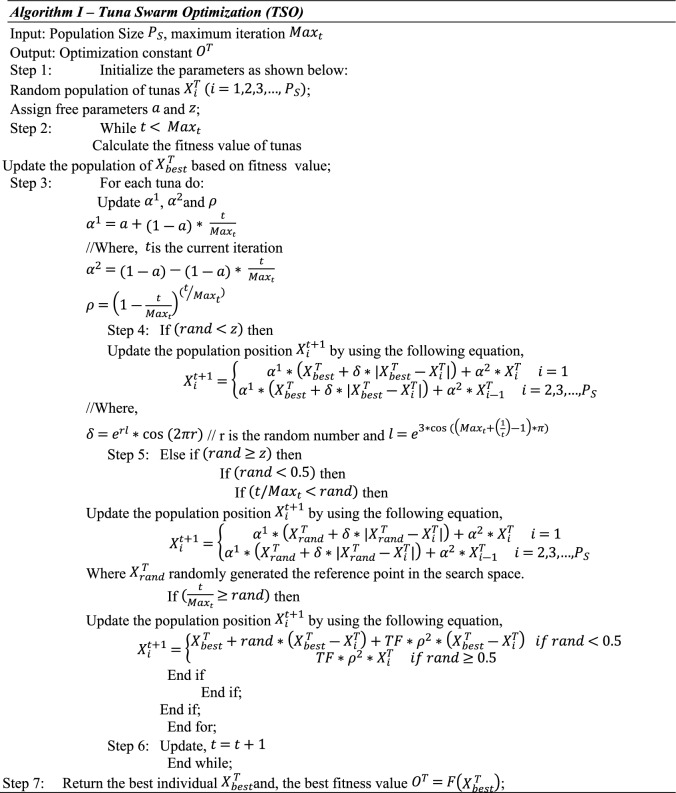


## Results and discussion

This section presents the performance analysis of the proposed security model concerning the measures of overall time cost, key generation cost, encryption time, decryption time, storage efficiency, computational efficiency, trust, entropy, precision, and storage overhead. To demonstrate the overall effectiveness and optimal performance rate of the suggested scheme, the acquired results are also compared with other current state-of-the-art models. A prototype model is implemented and evaluated for validation using the MIRACL library and the Python 3.7 tool. Moreover, the network is simulated for system implementation and validation, and its parameter specifications are shown in Table 3.

**Table 3:** System specifications.

Parameters	Specifications
No of cloud service providers	20
No of patients	100
Count of adversaries in patients	20
Count of adversaries in service providers	5
Transactions epochs	100
Trust value	0.6 to 1
Block prehash value	32
Time stamp	4
Nonce	4
Transaction root	32

**Figure 4:**
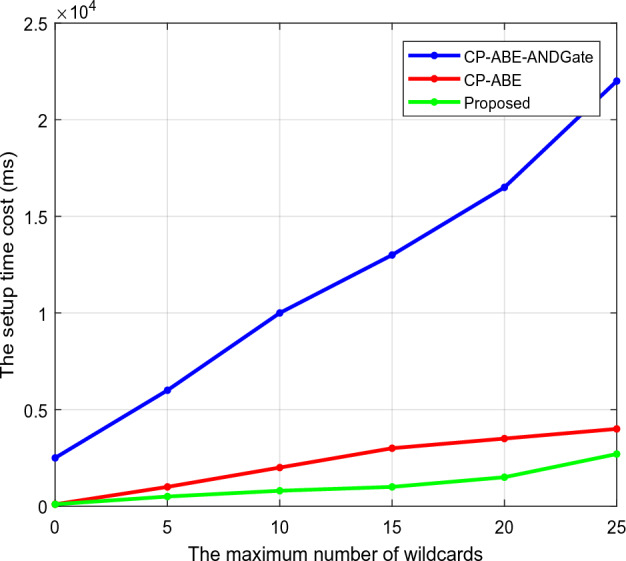
Setup time of existing and proposed security models.

**Figure 5:**
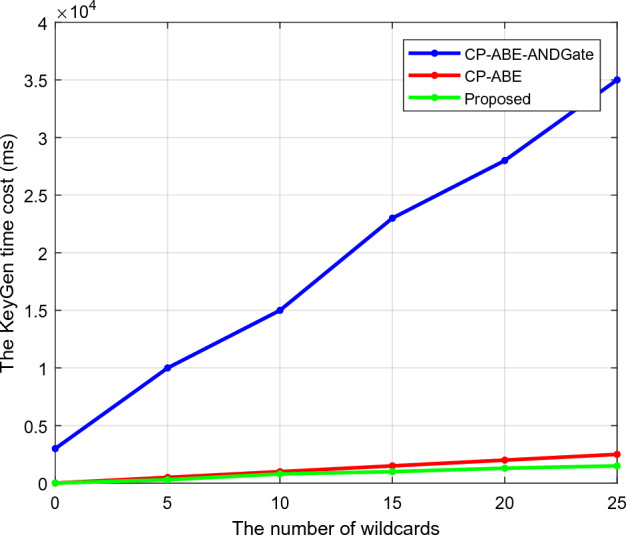
Key generation time.

Regarding the maximum number of wildcards, Fig. 4 estimates the setup time requirements of the existing Ciphertext-Attribute-Based Encryption (CP-ABE) integrated with AND gate and CP-ABE and proposed CTKGM-QTRAM security models. Here, the wildcard denotes the access structure's symbol (*), which is likewise regarded as a crucial component in cryptographic techniques. To calculate the setup time, this analysis determines the access policy's maximum allowed the number of wildcards. The setup time is typically described as the length of time the security model needs to initialize the setup phase before data transmission can begin. This analysis makes it clear that the suggested CTKGM-QTRAM technique requires less setup time (ms) than the other methods. Similarly, Fig. 5 calculates the estimated key generation times for traditional^30^ and suggested security models. One of the crucial steps in wireless data transmission and communication systems is key creation since the private and public key pair is crucial for securely sharing and retrieving data from the server. It is also determined by how long it takes the network management to generate these keys before data transmission and reception. The analysis demonstrates that the suggested CTKGM-QTRAM outperforms the traditional techniques with a quicker key generation process.

**Figure 6:**
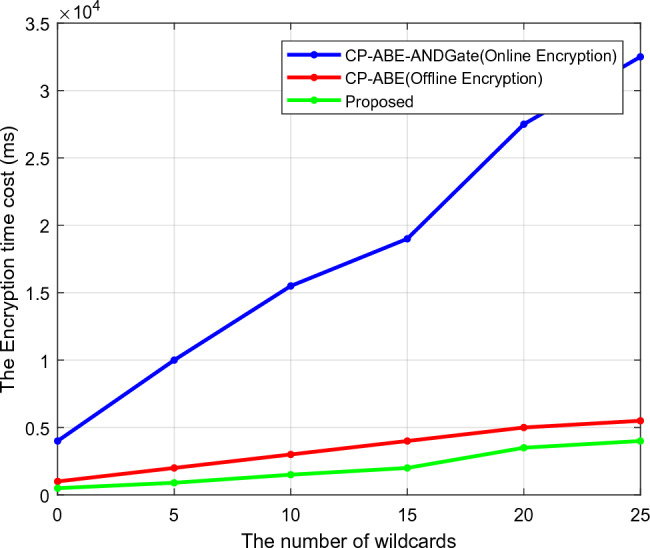
Encryption time.

**Figure 7:**
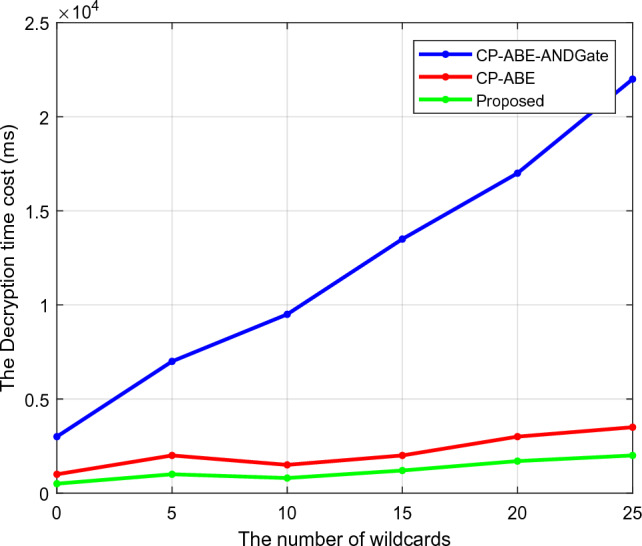
Decryption time.

Regarding the number of wildcards, Figs. 6 and 7 compare the encryption and decryption times of traditional and suggested security models. The time required by the security model to transform the original data into the cipher data is known as the encryption time. The decryption time determines the reverse process. The model is advocated for sending data encrypted and receiving it decoded. Based on the random key and unique certificate-generating techniques, the encryption and decryption times of the proposed model are effectively reduced. As a result, this analysis shows that the suggested model performs better than competing methods with shorter encryption and decryption times.

**Figure 8:**
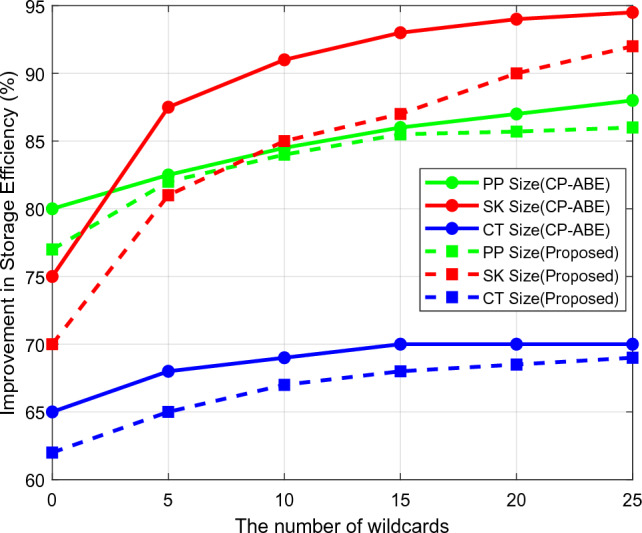
Storage efficiency.

**Figure 9:**
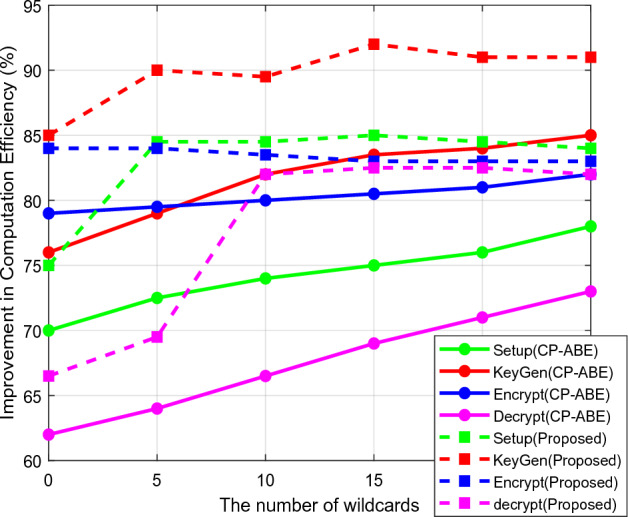
Computation efficiency.

**Figure 10:**
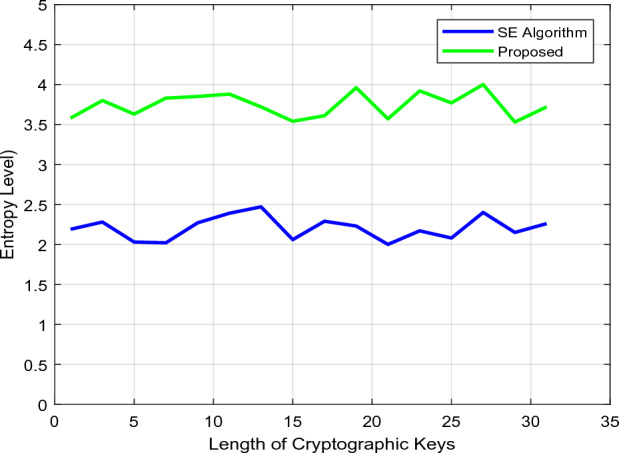
Entropy level.

**Figure 11:**
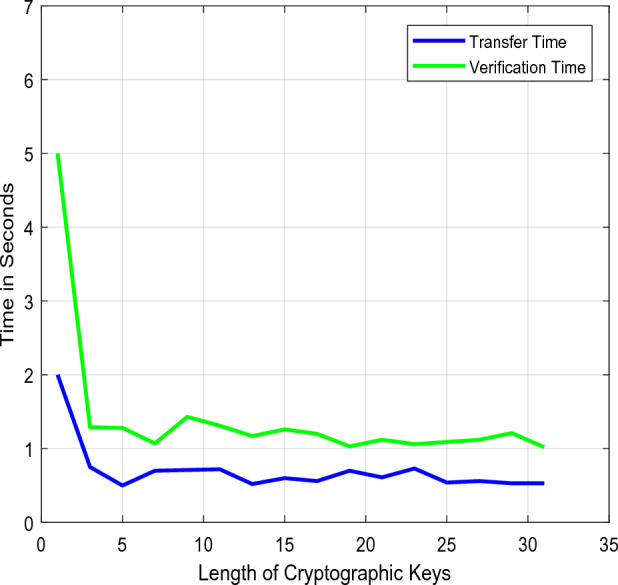
Transfer time and verification time.

The security models' storage and computing efficiency based on setup phase initialization, key generation, encryption, and decryption activities is compared in Figs. 8 and 9. Based on the number of operating steps, the overall storage and computational efficiency are examined in this case. The current methods typically involve more computations to boost data transfer security, which increases storage requirements and complicates computations. Concerning the variable length of cryptographic keys, Fig. 10 confirms the entropy level of existing and proposed approaches. The entropy level of a data sequence is typically calculated primarily for calculating uncertainty, which aids in the analysis of the unpredictability of public parameters. According to the computed outcomes, it can be seen that the suggested scheme's entropy is effectively raised for all key lengths. Furthermore, the data transfer and verification times are determined by the conventional standard^31^, and the suggested security models are evaluated concerning different cryptographic lengths, as shown in Fig 11.

**Figure 12:**
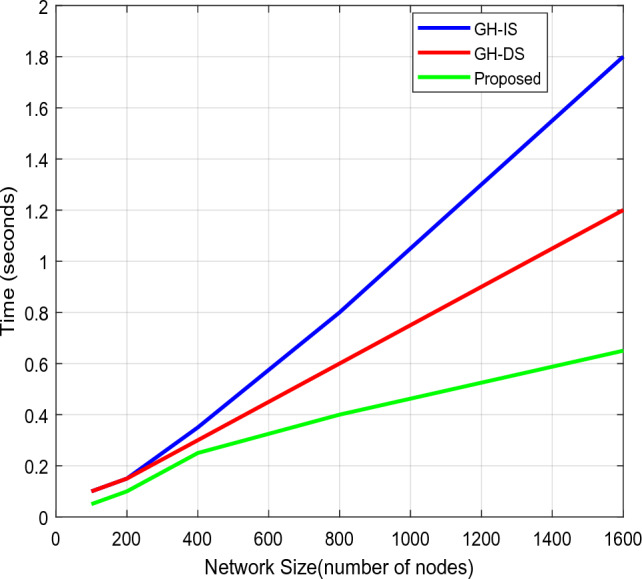
Overall time analysis.

Here, the transfer time refers to the period that the data is transmitted, and the verification time refers to the period that the receiving end spends validating the data. The findings demonstrate that the suggested approach necessitates a minimum amount of time for data transmission and verification procedures. The total amount of time needed to enable the safe data exchange and retrieval processes is next examined about the various participant counts, as shown in Fig. 12. Here, the Guard Health Information Sharing (GH-IS) and Guard Health Data Storage (GH-DS)^29^ methods are examined. The key renewal and validation processes add time to standard GH schemes for each transaction. As a result, the suggested strategy performs better than these methods with less time spent on computation and guaranteed data sharing security.

**Figure 13:**
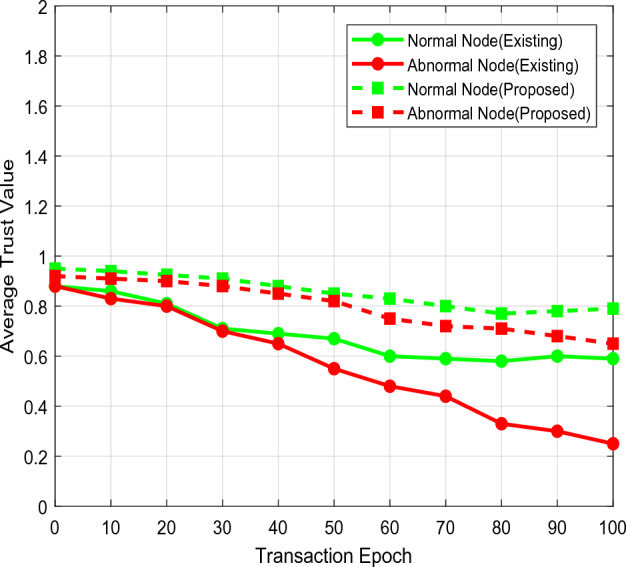
Average trust estimation.

The average trust value of current and proposed schemes is calculated in Fig. 13 and compared under various transaction epochs. By measuring the trust value with the use of BAN logic, the QTRAM protocol is primarily intended to enable data sharing between communication parties, such as patient users and healthcare servers. Furthermore, identifying the atypical users following the predicted trust value aids in maintaining the order of data transfer. As a result, the suggested QTRAM protocol outperformed the alternative method in terms of user confidence for both regular and abnormal users. For verifying the efficacy and detection effectiveness of security models, precision is one of the metrics that are most frequently employed in security applications.

**Figure 14:**
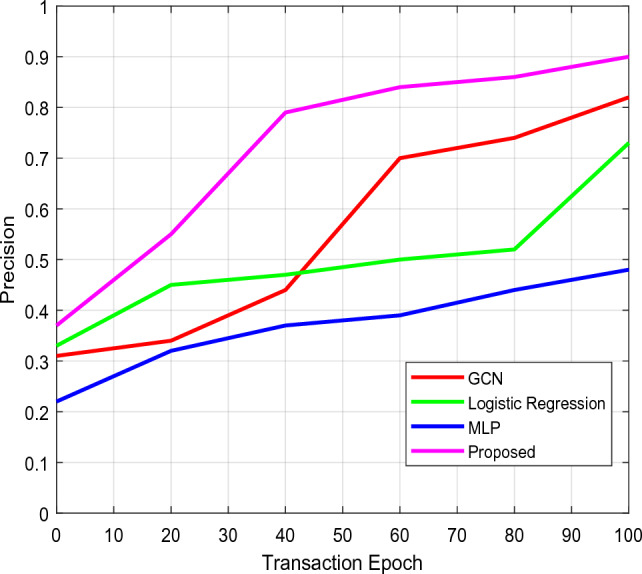
Precision Vs Transaction Epoch.

**Figure 15:**
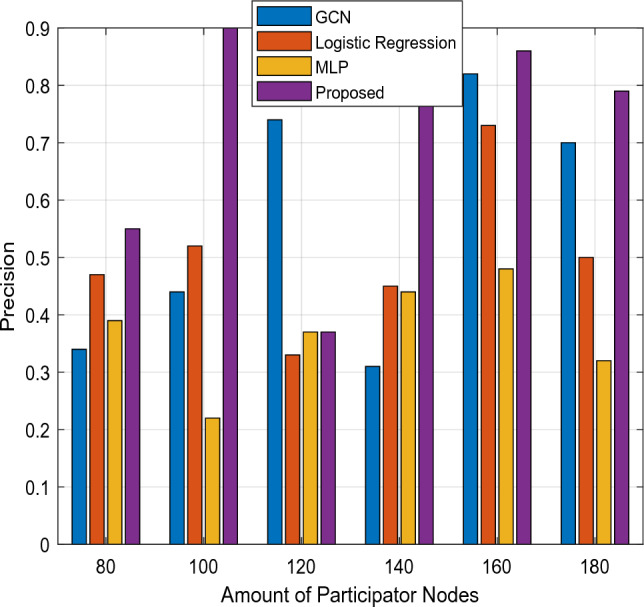
Precision Vs total number of participants.

**Figure 16:**
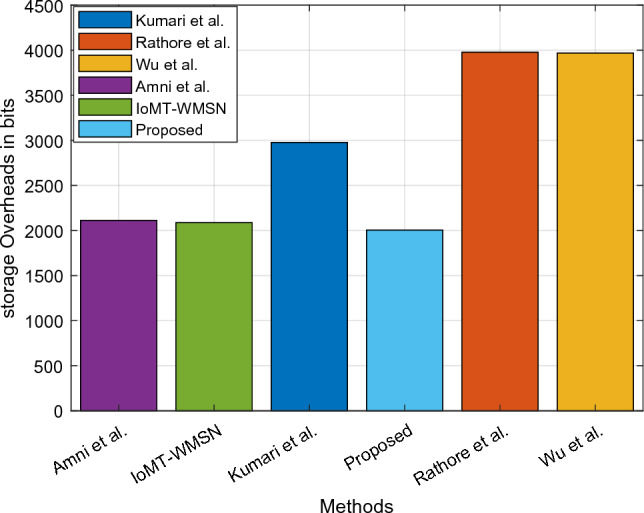
Storage overhead.

Here, Figs. 14 and 15 analyze the degree of accuracy for both traditional and novel security mechanisms concerning various transaction epochs and the overall participant count. According to the results, the suggested CTKGM-QTRAM scheme's accuracy is significantly higher than that of the other models, such as the Graph Convolutional Network (GCN), logistic regression, and Multilayer Perceptron. In Figure 13, the precision of existing GCN, LR, MLP techniques are compared with the proposed model with respect to varying transaction epochs. The experiment takes into account the methods LR, MLP, and GCN. After making numerous adjustments, we determined that our suggested model required two hidden layers with 100 neurons each, and it was trained for 500 epochs using the data transaction dataset with a learning rate of 0.001. Our detection model outperforms the other traditional classification algorithms, according to the precision results. In Figure 14, the precision is compared with respect to the amount of participation nodes from 80 to 180. As seen in this analysis, the detection accuracy increases as the number of participants rises. Additionally, we discovered that the proposed security approach heavily depends on the node's group size. The precision of proposed detection system is substantially better than the conventional MLP, LR, and GCN models. Additionally, as illustrated in Fig. 16, the storage overhead is calculated to determine how much space the security models require to store user data. The suggested approach uses blockchain technology to store the patient users' encrypted data in blocks with hash values, requiring only a small amount of server storage capacity. As a result, the storage complexity of the suggested scheme is effectively decreased when compared to the other approaches. Table 4 presents the comparative study of existing and proposed blockchain based security models used in the healthcare applications based on the security analysis. In this study, the different types of security parameters such as flexibility, availability, decentralized access, identity management, user authentication, privacy, and integrity have been considered. According to the findings, it is evident that the proposed blockchain could effectively satisfy all the security parameters for ensuring the reliable data transmission in the healthcare application systems.

**Table 4:** Security analysis.

Security properties	Blockchain healthcare 4.0^51^	Lightweight privacy model^52^	Blockchain based healthcare^53^	MeDShare^54^	Collaborative blockchain^55^	Proposed
Flexibility	X	X	X	X	X	✓
Availability	X	X	X	✓	✓	✓
Decentralized access	X	X	✓	✓	✓	✓
Identity Management	X	✓	X	✓	X	✓
User Authentication	✓	✓	✓	✓	X	✓
Integrity	✓	✓	✓	✓	✓	✓
Data Privacy	✓	✓	✓	✓	✓	✓

## Conclusion

This study created a sophisticated cybersecurity architecture to allow for safe data transmission in healthcare systems. Here, a variety of techniques are available for patient users to employ to exchange their private health information with the hospital domain securely. Patients are initially registered with the hospital server based on their personal information and medical records. For future communications, they get a special ID and certificate from the network manager. To generate the private and public key pair for the encrypted data before data sharing, the CTKGM is implemented after registration and is based on the generation of random hash values. By dividing the encrypted data into blocks of hash values, blockchain technology is primarily employed in this architecture to securely store the data in the server. Therefore, unauthorized users cannot access patients' private or confidential information. The QTRAM protocol is used to analyze the data receiver's confidence based on feedback inputs while the data is being transmitted. The BAN logic has been used in this procedure by calculating the trust score to verify the receiver's legitimacy. Also, the receiver-side nonce message is validated using the TSO algorithm, maintaining the collection of default messages. It offers the best value for sending the nonce message to them following the data request; hence, the validation has been completed. Here, security is guaranteed by the use of private and public key pairs for data encryption, blockchain technology for data storage, trust score estimate for safe transmission, and nonce message verification. To validate the effectiveness of this security architecture, several metrics including time cost, storage complexity, trust value, precision, and entropy have been computed. The suggested CTKGM- QTRAM-TSO methodology surpasses the alternative methods with better performance outcomes, as shown by a comparison of the results with other current state-of-the-art models. The main aim of this paper is to develop a new quantum blockchain based security framework to ensure the secure data transmission in healthcare system. For this purpose, we have used a reliable QTRAM and TSO in this work. Since, the contribution is to enable the secured data transmission in a networking system, so the suggested mechanism is suitable for some other networking applications that are incorporated with IoT, IIoT, or cloud systems.

This research can be expanded in the future by creating a sophisticated machine learning algorithm for categorizing the many kinds of hostile actions in the cybersecurity system.

## Data Availability

The datasets used and/or analysed during the current study are available from the corresponding author on request.
