# Author Correction: A quantum trust and consultative transaction-based blockchain cybersecurity model for healthcare systems

**DOI:** 10.1038/s41598-023-36573-8

**Published:** 2023-06-09

**Authors:** Shitharth Selvarajan, Haralambos Mouratidis

**Affiliations:** 1 Post Doc Researcher (IADS), University of Essex, Colchester, UK; 2 Department of Computer Science, Kebri Dehar University, Kebri Dehar, Ethiopia; 3 Institute for Analytics and Data Science (IADS), University of Essex, Colchester, England UK

Correction to: *Scientific Reports* 10.1038/s41598-023-34354-x, published online 02 May 2023

The original version of this Article contained an error in the name of the author Haralambos Mouratidis, which was incorrectly given as Haris Mouratidis.

The original Article has been corrected.

